# Psychological and Physiological Features Associated with Swimming Performance

**DOI:** 10.3390/ijerph18094561

**Published:** 2021-04-25

**Authors:** Vicente Javier Clemente-Suárez, Juan Pedro Fuentes-García, Ricardo J. Fernandes, João Paulo Vilas-Boas

**Affiliations:** 1 Faculty of Sport Science, European University of Madrid, 28670 Villaviciosa de Odón, Spain; vicentejavier.clemente@universidadeuropea.es; 2 Grupo de Investigación en Cultura, Educación y Sociedad, Universidad de la Costa, Barranquilla 11501-2060, Colombia; 3 Faculty of Sport Science, University of Extremadura, Avda. Universidad S/N, 10003 Cáceres, Spain; 4 Centre of Research, Education, Innovation and Intervention in Sport (CIFI2D), Faculty of Sport, University of Porto, Rua Plácido Costa, 91 4200 Porto, Portugal; ricfer@fade.up.pt; 5 Porto Biomechanics Laboratory (LABIOMEP), Faculty of Sport, University of Porto, Rua Plácido Costa, 91 4200 Porto, Portugal; jpvb@fade.up.pt

**Keywords:** autonomic nervous system, fatigue, maximal aerobic speed, sport performance

## Abstract

Background: The aim of the present research was to study the psychological and physiological features associated with aerobic and anaerobic performance in trained swimmers. Methods: A correlation and stepwise regression analyses were conducted with the data obtained in a RESTQ-76 sport questionnaire, a heart rate variability test, and an anaerobic and aerobic swimming performance efforts of 20 swimmers. Results: Aerobic performance correlated, principally, with parameters related to parasympathetic modulation measured in the frequency and time domains of the heart rate variability (LF/HF r: −0.806, *p* < 0.001; NN50 r: 0.937, *p* < 0.001). Swimmers’ anaerobic performance correlated to psychological features (low stress r: 0.526, *p*: 0.025, and high fatigue r: −0.506, *p*: 0.032). Conclusion: Swimming performance presented different psychological and physiological features depending on the probe characteristic. Specifically, swimmers’ anaerobic performance was associated with psychological features (low stress and high fatigue perception) and aerobic performance with physiological features (high parasympathetic modulation). This information could help coaches to know the variables to control in their swimmers, depending on the probe in which they compete (anaerobic or aerobic).

## 1. Introduction

Sport performance is the result of the interrelation of different physiological and psychological factors. The athletes’ functional state before competition could influence performance in competition. Therefore, the control of athletes’ physiological and psychological state, and the training conducted, are important factors to achieve a high performance [1]. However, the control of training performed is not always successful, especially when physical demands increase, increasing the external and internal workload that can produce a negative phase in the athletes’ adaptive process, affecting both performance and heath of athletes [2]. In these increased training periods, athletes may experience general fatigue, impaired performance, and states of overreaching may appear. A controlled overreaching could be positive for performance, but when this state lasts for several months, an overtraining syndrome is diagnosed [3].

Overtraining is one of the most commonly non-adaptative states of athletes, and they have been reported in different sport specialties during a competitive season [4,5]. The overtraining state has several detectable and measurable psychological implications that arise from the influence of both internal agents to training and competition, mistrust, frustration, fear, and external agents: social agents, educational agents, and individual agents [6]. This non-adaptative response of the organism affects the autonomic modulation of athletes, increasing the sympathetic and decreasing the parasympathetic modulation of the autonomic nervous system [7]. Currently, the heart rate variability (HRV) is used as a training control method and overtraining state diagnosis, being a useful tool to assess the athletes’ adaptation state [8].

An adaptative response of the autonomic nervous system is associated with a higher sport performance [9], and it is also influenced by both physiological factors, such as the training sessions performed, and psychological factors, such as emotional state and stress levels of the athletes [10]. When the adaptive capacity of the athlete is exceeded, it cannot continuously perform the training, and performance decreases. Several psychological instruments have been developed to evaluate the psychological state of athletes, evaluating states of anxiety, the positive and negative affect scales, or the profile of mood states [11], and more specifically, the recovery–stress questionnaire for athletes (RESTQ-76 Sport) evaluates physical stress, mental stress, and recovery strategies of the athlete [12].

Several authors hypothesized that the control of athletes’ psychological and physiological status is an important factor to achieve the highest performance, to avoid overreaching, overtraining, and non-adaptative response to training [8,11,13]. There are specific organic responses to training related to higher performance as an increase in the autonomic modulation, a higher parasympathetic domain modulation, higher recovery perception, and smaller fatigue perception of athletes [8,9,10], but there are general recommendations that do not take into account the specific characteristic and psycho-physiological demands of the different sports. According to the physiological demands, cycle sports could be divided in aerobic and anaerobic disciplines, being the metabolic, neuromuscular, and psychological demands different. Then the psycho-physiological profile of athletes to achieve the highest aerobic or anaerobic performance would be different due to the different requirements and psycho-physiological demands of these efforts.

Traditionally, high volume low intensity training (linear periodization, block periodization) is associated with sport performance, but the physiological consequences of these eliciting training produce hyperactivation of autonomous sympathetic nervous system as well as non-adaptative psychological modifications (anxiety, emotive disruption, demotivation…). Actually, a new training paradigm based on low volume high intensity (reverse periodization) produce same or higher performance as well as parasympathetic modulation and no negative effects on psychological profile of athletes [8,11]. To the best of our knowledge, no research has studied the association between the physiological and psychological profile of swimmers and performance in aerobic and anaerobic efforts. For this reason, the aim of the present research was to study the psychological and physiological features. associated with aerobic and anaerobic performance, in trained swimmers. It was hypothesized that the psychological and physiological profile related to the higher performance in aerobic and anaerobic efforts would be different because of the different psychological and physiological demands of each effort.

## 2. Materials and Methods

### 2.1. Participants

Twenty trained swimmers were analyzed, 9 females and 11 males. The characteristics of the swimmers were: weight M = 67.3, SD = 11.4 kg; height M = 1.80, SD = 0.1 m; BMI M = 21.5, SD = 2.3 m/kg^2^; age M = 18.1, SD = 2.8 years. Swimmers had a mean of 6.5 years of training experience, and all of them competed at national level. They competed in swimming proof from 50 to 400 free style probes. The inclusion criteria was: (i) competing at national level, (ii) At least 5 years of swimming training experience, (iii) no injuries present, (iv) not taking any medications. The population that reached the inclusion criteria were 95 swimmers, then the sample size obtained presented a confidence level of 90% and a 16% of margin of error between. Prior to participation, the experimental procedures were explained to all participants, who gave their voluntary written informed consent and understood that they were free to withdraw from the study at any time. The study was designed in compliance with the recommendations for clinical research of the Declaration of Helsinki of the World Medical Association. The protocol was reviewed and approved by the local ethics committee.

### 2.2. Study Design

In order to reach the study aims, participants were instructed to have a high carbohydrate meal the previous day before the evaluation days, as well as to drink at least 2 L of liquid (preferably water). In this line, the 2 days prior to the evaluation day, swimmers conducted only light training, 30–40 min aerobic swimming. The experimental evaluation was conducted between 09:00 to 12:00 AM. The evaluations were conducted at the beginning of the season in October.

### 2.3. Procedure

A correlation study was performed. The inclusion criteria to select the participants were: compete in national trial, train more than five days per week, and had a minimal experience in swimming training and competition of 4 years, in order to avoid possible training adaptation due to the lack of training experience. The sampling type selected was intentional because of the difficulty to obtain participants for the study. Participants completed the same day a RESTQ-76 Sport questionnaire to analyze psychological features, a HRV test to analyze physiological features, and a 50-m maximum swimming test (anaerobic effort). The day after, they performed an incremental maximum swimming test (aerobic effort).

#### 2.3.1. Psychological Measures

The RESTQ-76 Sport is a psychometric instrument that can be used to assess individuals’ recovery-stress state. The test-retest reliability of RESTQ-76 Sport has previously been reported (0.7) [14]. The Portuguese version of the RESTQ-76 sport, previously traduced and validated [15], was used in the present study. The questionnaire uses a self-report approach to evaluate physical, subjective, behavioral, and social aspects of stress and recovery. Within each scale, the swimmer must respond to four specific items. The items are then rated according to their frequency on the Liker scale, ranging from 0 (never) to 6 (always), which indicates how often the athlete has participated in various activities during the last 72 h. The questionnaire consisted of 77 items divided into 19 basic scales (10 stress subscales and 9 recovery subscales), with four second order dimensions as follows: 7 not sport-specific stress (NSSS) scales: general stress, emotional stress, social stress, conflicts, fatigue, lack of energy, and physical complaints; 5 not sport-specific recovery (NSSR) scales: success, social recovery, physical recovery, being in shape, and sleep quality; 3 sport-specific stress (SSS) scales: disturbed breaks, emotional exhaustion, fitness-injury; 4 sport specific recovery (SSR) scales: self-efficacy, self-regulation, personal accomplishment, and general well-being. From the second order, dimensions can be achieved with the overall dimensions of stress (TS) and recovery (TR).

While this method of analysis is not included in the original RESTQ-76 Sport Manual [12], previous research has provided concrete evidence for its validity as a practical measure for assessing global changes in the stress recovery balance in athletes [16,17]. We analyzed the following variables: (i) general stress, (ii) emotional stress, (iii) social stress, (iv) conflict, (v) fatigue, (vi) lack of energy, (vii) physical complaints, (viii) disturbed breaks, (ix) emotional exhaustion, (x) fitness-injury, (xi) success, (xii) social recovery, (xiii) physical recovery, (xiv) general well-being, (xv) sleep quality, (xvi) being in shape, (xvii) personal accomplishment, (xviii) self-efficacy, (xix) self-regulation, (xx) not sport-specific recovery, (xxi) not sport-specific stress, (xxii) sport-specific stress, (xxiii) sport specific recovery, (xxiv) total stress, (xxv) total recovery, and (xxvi) recovery-stress ratio (R-S ratio).

#### 2.3.2. Physiological Measures

HRV test was performed to evaluate swimmers’ autonomic nervous system state. HRV test lasted for 10 min in supine position, lying in a stretcher in a room with controlled temperature, just as previous research [18,19]. The RR interval (time between two R waves of the recorded cardiac electrical activity) was measured with a Polar S810 heart rate monitor (Polar, Kempele, Finland) that showed a good agreement with HRV determined from electrocardiogram [20]. The RR series were analyzed using a Kubios HRV software (version 2.0, Biosignal Analysis and Medical Imaging Group, University of Kuopio, Kuopio, Finland) which was developed in accordance with published recommendations [21]. The software showed excellent validity [22] and is able to account for non-linear trends, often present in beat-to-beat recordings, by detrending the filtered RR data using the smoothness prior approach [22]. Data were interpolated at a rate of 4 Hz in accordance with the software’s recommendations [23].

We analyzed the following variables of the HRV frequency and time domains: (i) total power, (ii) the very low frequency band (VLF), (iii) the low frequency band (LF), (iv) the high frequency band (HF), (v) the normalized LF and HF, (vi) the LF/HF ratio, (vii) the percentage of differences between adjacent normal RR intervals more than 50 ms (pNN50), (viii) the number of differences between adjacent normal RR intervals higher than 50 ms (NN50), (ix) the standard deviation of all normal RR intervals (SDNN), (x) the square root of the mean of the sum of the squared differences between adjacent normal RR intervals (RMSSD), (xi) mean heart rate (HR), and (xii) mean RR.

#### 2.3.3. Performance Measures

Anaerobic and aerobic swimming efforts were performed in a 25-m indoor swimming pool. The temperature of the air in the swimming pool was 29 °C, the humidity was 47%, and the temperature of the water was 27.5 °C. All swimmers were familiarized with the test and instruments used in this study. The anaerobic effort was a 50-m maximum swimming test. Time to complete the 50 m maximum test was measured through a chronofrequencemeter (Seiko, 5141). The day after the 50 m maximum swimming tests, the aerobic effort was conducted, each participant performed a 5 × 200 m individualized intermittent incremental protocol, with 30 s of rest period between steps. Researchers and coaches defined the velocity of the last step of the protocol through the 400 m front crawl best time that swimmers were able to accomplish at that moment (using start in-water and open turns). Then, 4 successive 0.05 m/s were subtracted from the swimming velocity, corresponding to the last step, allowing the determination of the mean target velocity for each step [24]. Swimming velocity was measured through a chronofrequencemeter, being controlled through auditory signals each 25 m. Oxygen uptake was measured through direct oximetry by a telemetric portable gas analyzer (K4b2, Cosmed, Rome, Italy) connected to the swimmer by a respiratory snorkel and valve system (AquaTrainer II Snorkel^®^, Cosmed, Rome, Italy). HR was monitored and registered continuously every 5 s through a heart rate monitor system (Polar Vantage NV, Polar lector Oy, Kempele, Finland) that telemetrically emitted the data to the K4b2 portable unit. Maximal oxygen uptake (VO2max) was considered to be reached according to traditional physiological criteria [25]: (i) occurrence of a plateau despite an increase in swimming velocity, (ii) high levels of blood lactate concentration (≥8 mmol/l), (iii) elevated respiratory exchange ratio (r ≥ 1.0), (iv) elevated HR (≥90% of [220-age]), and (v) an exhaustive perceived exertion, controlled visually and case by case. Maximal aerobic speed (MAS) was determinate for each swimmer, MAS is defined as the minimum speed which elicits VO_2_max and is considerate as an important aerobic performance predictor [26]. We analyzed the following performance variables: (i) time in 50 m swimming test, (ii) rate of perceived exertion, Borg 6–20 level scale (RPE), in 50 m swimming test, (iii) MAS, and (iv) RPE at MAS (RPE-MAS).

### 2.4. Statistical Analysis

To analyze the data SPSS statistical package (version 17.0; SPSS, Inc., Chicago, IL, USA) was used. The Shapiro–Wilk normality test was used to test homogeneity of each variable. Then a bivariate correlation analysis between the psycho-physiological and performance parameter were performed using Pearson correlation analysis. To analyze which physiological and psychological features were the best predictor of aerobic and anaerobic performance, a stepwise regression with T50 m RPE-50, MAS, and RPE-MAS variables as the dependent variables, and the physiological and psychological data as predictors were used. The level of significance for all the comparisons was set at *p* < 0.05.

## 3. Results

Data are reported as mean ± SD. Values measured in the RESTQ-76 sport test are showed in Table 1. We found how swimmers presented higher values in the total recovery than in the total stress second order scales.

The values obtained in HRV frequency and time domains, and values of aerobic and anaerobic performance are showed in Table 2. There was a higher values of high frequency band in the frequency domain analysis, as well as high values in the different time domain analysis parameters.

The bivariate correlation analysis presented a different relationship between psychological and performance variables (Table 3). The anaerobic performance (T50) positively correlated to general stress, emotional stress, and social stress and negatively correlated to fatigue, personal accomplishment, self-regulation, physical recovery, and sleep quality. Aerobic performance (MAS) positively correlated to disturbed breaks, personal accomplishment, sleep quality, and fitness injury.

The physiological parameters analyzed presented a different relationship with performance, showing time domain parameters a positive correlation with aerobic performance (MAS) and different correlations in the frequency domain of HRV (Table 4).

Finally, the stepwise regression analysis showed the relation between performance in the aerobic and anaerobic test with HRV variables (Table 5). The best predictor of both aerobic and anaerobic performance, after the realized stepwise regression analysis, were the VLF, NN50, social recovery, physical recovery, and R-S ratio.

## 4. Discussion

The aim of the present research was to study the psychological and physiological features associated with aerobic and anaerobic performance in trained swimmers. Results obtained showed a different relation between psychological and physiological features of swimmers and aerobic and anaerobic performance. The initial hypothesis was compiled, once the higher aerobic and anaerobic performances were related, with different psycho-physiological profiles.

### 4.1. Psychological Features

Values obtained in stress subscales of the present research swimmers were higher than those obtained during rest and training periods in volleyball players; however, the recovery perception of swimmers was smaller than in rest periods but similar in the training period of volleyball players [27]. Then, the perceived stress associated to the training workload of swimmers was higher than volleyball players. Values measured in swimmers were consistent with the results obtained in tennis players after 16 weeks of training, except general stress and conflicts, which were higher in tennis players than in swimmers, revealing a higher perceived stress in tennis player than in swimmers during training, probably due to tennis being an opposition sport and the higher stress of competitions [28].

Performance in the anaerobic effort presented more correlation with stress subscales than performance in the aerobic effort (anaerobic effort: general, emotional, social stress and fatigue vs. aerobic effort: disturbed breaks and fitness-injury, respectively). High stress values (general, social, and emotional) adversely affect performance in the 50 m maximal swimming test, and emotional stress especially presented the higher correlation. It shows, again, the importance of a non-stressful environment on performance [29], especially in anaerobic swimming efforts. By contrast, fatigue presented an inverse moderate correlation with anaerobic performance. Therefore, a fatigue perception of swimmers is associated with less time in the 50 m maximal swimming test. This could be explained since swimmers that performed more training would be more fatigued, but they could achieve a better time in the 50 m maximal swimming test because of this higher training volume. This result is opposite to results obtained by triathletes in an aerobic performance test (3 km running test) [16], and it is also contrary to the result obtained by Rossi, McMillan, and Buckley [13] that found a decrease in drop jump height and flight time related to an increase in perceived stress and reduced total recovery-stress scores. Therefore, it seems that fatigue perception adversely affects aerobic and explosive strength efforts, but not anaerobic efforts such as the 50 m maximal swimming test.

In relation to recovery, this refers not only the absence of stress, also the restoring processes of psycho-physiological resources and their appropriate reserves. RESTQ-76 Sport recovery subscale of swimmers presented satisfactory values since no cases of extreme fatigue were found. For these situations, and to improve the fatigue-recovery state, Stefanello [30] successfully used cognitive techniques such as self-report, awareness or concentration and somatic techniques through breath control. It is important to consider that the recovery is an individual process that occurs over the time and which depends on the type and duration of the stressor agent. Therefore, the recovery is complete only with a psycho-physiological state that could restore the homeostatic balance [2]. A higher number of recovery subscales were related to performance in anaerobic than in aerobic efforts (4 vs. 2 respectively), also general recovery factors (NSSR) as well as total values of recovery (TR) showed a significantly correlation with anaerobic performance. By contrary aerobic effort did not correlate to any general scales. Therefore, the swimmer’s recovery perception presented a higher influence in anaerobic than in aerobic performance efforts. Personal accomplishment and sleep quality (recovery subscales) have been the unique variables related to both anaerobic and aerobic performance, but showing an opposite relationship, negative in anaerobic effort and positive in aerobic effort.

The RPE of swimmers in the anaerobic effort presented a positive correlation with the recovery subscales, while stress subscales showed a negative relation. A similar trend was observed in general scales, also showing a high positive correlation between R-S ratio and RPE in the anaerobic effort. It is evident, while fatigue perception was lower and recovery perception was higher, the RPE in the anaerobic effort was lower. By contrast in the aerobic effort the results obtained were completely opposed. The stress subscale presented a moderate positive correlation with the RPE, while recovery subscales was negatively correlated to RPE. In relation to the general scale, only recovery values (total recovery, not sport specific recovery and sport specific recovery) obtained a moderate negative correlation with the RPE values. So, the psychological factors analyzed in the RESTQ-76 sport questionnaire presented an inverse tendency in the relationship with the RPE in the anaerobic and the aerobic efforts, showing recovery subscales a negative correlation with anaerobic performance and a positive correlation with aerobic performance.

### 4.2. Physiological Features

HRV data showed different correlations with RPE depending on the effort performed. In the aerobic effort, higher sympathetic activation was correlated to higher RPE. This result might be related to the association between sympathetic activation and low performance and overtraining symptoms [8]. It is interesting to link these results to other stressful water situations such as underwater evacuation training, which produce an increase in the sympathetic nervous system modulation, elevating the psycho-physiological stress response [31,32]. By contrast, in the anaerobic effort, results were contradictory because both higher sympathetic and parasympathetic activation were related to lower RPE. This could be explained by increases in the autonomic modulation, which means an increase in the activation levels of the two divisions of autonomic nervous system, sympathetic and parasympathetic, were previously associated with increased athletic performance [33]. Moreover, regarding the performance variables analyzed, the aerobic performance (MAS) was highly correlated to both frequency (HF, LF/HF, LFn and HFn) and time domains (Mean RR, Mean HR, NN50 and pNN50), by contrast, the anaerobic performance (T50m) correlated only with two variables of the frequency domain (LF and HF). Specifically, higher aerobic performance was related to a higher parasympathetic activation because athlete who presented higher values of HF, HFn, LF/HF, Mean RR, NN50 and pNN50 achieved a higher MAS, results coinciding with previous studies [34,35,36] conducted with athletes. However, anaerobic performance correlated to lower values of LF and HF, it seems that the activation of the autonomic nervous system appears to be not a parameter that highly influences the anaerobic performance. Therefore, we found that the physiological state of autonomic nervous system was more related to aerobic performance than with anaerobic performance, specifically a higher parasympathetic activation, was more related to performance. Finally, in the stepwise regression analysis variables of VLF, NN50, social and physical recovery, and R-S ratio were significantly related to performance in both aerobic and anaerobic efforts. These results highlighted the importance of recovery, not only physically, but also how the influence of social environment could affect athletes’ performance [37,38].

In the multidisciplinary construct of athlete’s health and performance, new variables are adding to this complex scenario. In this line, the bioelectric phase angle has been presented as an ecological approach to the swimmers’ health and condition assessment [39]. Specifically, regarding the training periodization, previous research encourages coaches to consider that athletes might be affected by the specific recovery time of previous exercise performed, also suggesting that the management of the exercise intensity and appropriate monitoring of cardiac autonomic parameters might be helpful to know the physical condition of them [40].

## 5. Practical Application

Training monitorization of different psychological and physiological parameters to avoid overreaching and overtraining syndromes has been commonly used in competitive sports such as swimming. A general recommendation of the correct athletes’ psycho-physiological status is used to conduct this monitorization process, disregarding the specific psycho-physiological profile of different anaerobic and aerobic probes. It is known that the psycho-physiological demands of different anaerobic and aerobic probes, but to the best of our knowledge, the relation between them and the swimmers’ performance is poor knowledge. The present research showed the specific psychological and physiological parameters related to anaerobic and aerobic performance. Thereby, performance in an anaerobic swimming effort was related to more psychological parameter than an aerobic swimming effort, and conversely, performance in an aerobic effort was more related to physiological parameter than the anaerobic effort. Specifically, anaerobic swimming performance was adversely affected by high stress values in general, social, and emotional scales, but a higher value of fatigue perception was positively related to performance in this effort. Related to physiological variables, anaerobic swimming performance correlated to low LF and HF values of the frequency domain of the heart rate variability. The aerobic swimming performance was related principally with a higher parasympathetic modulation of the autonomic nervous system, reflected in high values of HF, HFn, Mean RR, NN50, and pNN50 and low values of LF, LFn, LF/HF, and Mean HR.

Data obtained in the present research could be used by coaches to improve monitorization actions and focus on specific parameters depending on the swimmer’s competition probe. In this line, psychological monitorization of stress in aerobic performance swimmers could be easily applied using the RESTQ-76 Sport questionnaire and implementations of psychological techniques such as mindfulness or meditation could be applied to decrease possible increased stress moments. The use of HRV monitorization could help coaches to modify training loads according to the swimmer’s specialty profile showed in the present research.

## 6. Study Limitations

The low number of swimmers and the no control of biochemical stress related variables (as cortisol, alpha amylase…) were a limitation of the study. The inclusion criteria and financial lacks precluded these aims. Additionally, the non-randomized order of experimental trials was a concern. The effects of wearing a gas analyzer during the aerobic and anaerobic performance test could be also considered a limitation, since swimmers did not swim in real conditions, but it is actually the only way to analyze gas exchange in swimming.

## 7. Conclusions

Swimming performance presented different psychological and physiological features depending on the probe characteristic. Specifically, swimmers’ anaerobic performance was associated with psychological features (low stress and high fatigue perception) and aerobic performance with physiological features (high parasympathetic modulation). This information could help coaches to know the variables to control in their swimmers, depending on the probe in which they compete (anaerobic or aerobic).

## Figures and Tables

**Table 1:** Mean and standard deviation of values obtained in the RESTQ-76 Sport.

Scales	Measure	Value	Scales	Measure	Value
Stress Subscales	General Stress	1.8 ± 1.2	Recovery Subscales	Being in shape	2.9 ± 1.2
	Emotional Stress	2.0 ± 1.1		Personal accomplishment	3.9 ± 0.5
	Social stress	1.6 ± 1.1		Self-efficacy	3.3 ± 1.0
	Conflict	2.9 ± 1.4		Self-regulation	4.3 ± 1.1
	Fatigue	3.3 ± 1.0		Social recovery	3.5 ± 1.4
	Lack of energy	2.0 ± 0.6		Physical recovery	3.0 ± 0.9
	Physical Complaints	2.0 ± 0.6		General well-being	4.0 ± 1.1
	Success	3.6 ± 0.7		Sleep quality	2.8 ± 0.8
	Disturbed Breaks	2.8 ± 0.8			
	Emotional Exhaustion	1.7 ± 1.1			
	Fitness-injury	3.3 ± 0.9			
Second order scales	NSSR	2.3 ± 0.9			
	NSSS	3.4 ± 0.6			
	SSS	2.6 ± 0.5			
	SSR	3.6 ± 0.7			
	TS	2.4 ± 0.6			
	TR	3.5 ± 0.6			
	R-S ratio	1.1 ± 0.6			

NSSR—not sport specific recovery, NSSS—not sport specific stress, SSS—sport-specific stress, SSR—sport specific recovery, TS—total stress, TR—total recovery, R-S ratio—recovery-stress ratio.

**Table 2:** Mean and standard deviation of heart rate variability and performance variables.

	Variable	Value		Variable	Value
HRV Frequency Domain	TP (ms^2^)	12166.2 ± 3233.6	HRV Time Domain	Mean RR (ms)	939.5 ± 112.0
	VLF (ms^2^)	3286.5 ± 1422.2		SDNN (ms)	120.0 ± 28.5
	LF (ms^2^)	3459.5 ± 1422.2		Mean HR (bpm)	65.6 ± 7.8
	HF (ms^2^)	5419.9 ± 2307.6		RMSSD (ms)	134.5 ± 42.2
	LF/HF	0.69 ± 0.27		NN50 (count)	253.9 ± 105.5
	LFn (n.u.)	39.9 ± 7.9		pNN50 (%)	43.9 ± 22.7
	HFn (n.u.)	60.1 ± 7.9			
Anaerobic Performance	T50 m (s)	28.9 ± 2.1	Aerobic Performance	MAS (m.s^−1^)	1.30 ± 0.10
	RPE-50 m	15.9 ± 1.5		RPE-MAS	18.1 ± 1.9

T50—time in 50 m swimming test, RPE-50 m—rate of perceived exertion in 50 m swimming test, MAS—maximal aerobic speed, RPE-MAS—rate of perceived exertion at maximal aerobic speed, TP—total power, VLF—very low frequency, LF—low frequency, HF—high frequency, LFn—low frequency normalized, HFn—high frequency normalized, SDNN—standard deviation of all normal RR intervals, RMSSD—square root of the mean of the sum of the squared differences between adjacent normal RR intervals, NN50—number of differences between adjacent normal RR intervals more than 50 ms, pNN50—percentage of differences between adjacent normal RR intervals more than 50 ms, n.u.—normalized unit, ms—milliseconds, bpm—beat per minute.

**Table 3:** Correlation between stress, recovery RESTQ-76 Sport subscales and aerobic and anaerobic performance parameters. R and *p* values in parentheses.

	Measure	T50	RPE-50 m	MAS	RPE-MAS
Stress Subscales	General Stress	0.526 (0.025)	−0.711 (0.001)		
	Emotional Stress	0.911 (<0.001)			
	Social stress	0.629 (0.005)			
	Fatigue	−0.506 (0.032)	−0.738 (<0.001)		0.590 (0.010)
	Lack of energy		−0.789 (<0.001)		0.528 (0.024)
	Physical Complaints		−0.789 (<0.001)		0.528 (0.024)
	Disturbed Breaks		−0.570 (0.014)	0.555 (0.017)	
	Emotional Exhaustion				
	Fitness-injury			0.887 (<0.001)	
Recovery Subscales	Being in shape		0.676 (0.002)		−0.561 (0.015)
	Personal accomplishment	−0.669 (0.002)	0.715 (0.001)	0.477 (0.045)	−0.489 (0.039)
	Self-efficacy		0.894 (<0.001)		−0.836 (0.000)
	Self-regulation	−0.659 (0.003)			
	Social recovery		0.723 (0.001)		−0.993 (<0.001)
	Physical recovery	−0.490 (0.039)			
	General well-being		0.747 (<0.001)		−0.603 (0.008)
	Sleep quality	−0.891 (<0.001)		0.495 (0.037)	
General scales	NSSS		−0.713 (0.001)		
	NSSR	−0.661 (0.003)	0.645 (0.004)		−0.497 (0.036)
	SSS		−0.482 (0.043)		
	SSR		0.682 (0.002)		−0.543 (0.020)
	TS		−0.686 (0.002)		
	TR	−0.556 (0.016)	0.686 (0.002)		−0.538 (0.021)
	R-S ratio		0.914 (<0.001)		

T50—time in 50 m swimming test, RPE-50 m—rate of perceived exertion in 50 m swimming test, MAS—maximal aerobic speed, RPE-MAS—rate of perceived exertion at maximal aerobic speed, NSSR—not sport specific recovery, NSSS—not sport specific stress, SSS—sport-specific stress, SSR—sport specific recovery, TS—total stress, TR—total recovery, R-S ratio—recovery-stress ratio.

**Table 4:** Correlation between heart rate variability frequency and time domains and aerobic and anaerobic performance parameters. R and *p* values in parentheses.

	Variable	T50	RPE-50 m	MAS	RPE-MAS
Frequency Domain	TP (ms^2^)				
	VLF (ms^2^)				
	LF (ms^2^)	−0.684 (0.002)	−0.590 (0.010)		0.663 (0.003)
	HF (ms^2^)	−0.799 (<0.001)	−0.472 (0.048)	0.644 (0.004)	
	LF/HF			−0.806 (<0.001)	0.755 (<0.001)
	LFn (n.u.)			−0.774 (<0.001)	0.775 (<0.001)
	HFn (n.u.)			0.774 (<0.001)	−0.775 (<0.001)
Time Domain	Mean RR (ms)		−0.591 (0.010)	0.815 (<0.001)	
	SDNN (ms)				
	Mean HR (bpm)		0.545 (0.019)	−0.848 (0.000)	
	RMSSD (ms)		−0.483 (0.042)		
	NN50 (count)			0.937 (<0.001)	
	pNN50 (%)			0.884 (<0.001)	

T50—time in 50 m swimming test, RPE-50 m—rate of perceived exertion in 50 m swimming test, MAS—maximal aerobic speed, RPE-MAS—rate of perceived exertion at maximal aerobic speed, TP—total power, VLF—very low frequency, LF—low frequency, HF—high frequency, LFn—low frequency normalized, HFn—high frequency normalized, SDNN—standard deviation of all normal RR intervals, RMSSD—square root of the mean of the sum of the squared differences between adjacent normal RR intervals, NN50—number of differences between adjacent normal RR intervals more than 50 ms, pNN50—percentage of differences between adjacent normal RR intervals more than 50 ms, n.u.—normalized unit, ms—milliseconds, bpm—beat per minute.

**Table 5:** Stepwise regression analysis.

			VLF	NN50	Social Recovery	Physical Recovery	R-S Ratio
T50	Unstandardized Coefficients	B	<0.001	−0.009	2.257	−1.617	−2.004
		SE	2.462	7.052	1.277	1.282	1.241
	Standardized Coefficients	B	0.238	−5.627	0.841	−5.389	−7.699
RPE-50 m	Unstandardized Coefficients	B	<0.001	−0.001	0.667	0.002	1.053
		SE	<0.001	<0.001	<0.001	<0.001	<0.001
	Standardized Coefficients	B	0.184	−0.144	0.431	0.001	0.701
MAS	Unstandardized Coefficients	B	<0.001	0.001	−0.038	0.020	0.045
		SE	<0.001	<0.001	<0.001	<0.001	<0.001
	Standardized Coefficients	B	−0.141	1.045	−0.252	0.118	0.313
RPE-MAS	Unstandardized Coefficients	B	<0.001	−0.004	−2.228	0.324	−0.251
		SE	<0.001	<0.001	<0.001	<0.001	<0.001
	Standardized Coefficients	B	−0.240	−0.258	−0.896	−0.116	−0.104

R = 0.894, R^2^ = 0.798, adjusted R^2^ = 0.750, standard error of estimate = 23.063, F = 16.498, *p* ≤ 0.001, T50—time in 50 m swimming test, RPE-50 m—rate of perceived exertion in 50 m swimming test, MAS—maximal aerobic speed, RPE-MAS—rate of perceived exertion at maximal aerobic speed, VLF—very low frequency, NN50—number of differences between adjacent normal RR intervals more than 50 ms, R-S ratio—recovery-stress ratio.

## Data Availability

All study data are in the manuscript.
